# Evolution of a polymodal sensory response network

**DOI:** 10.1186/1741-7007-6-52

**Published:** 2008-12-15

**Authors:** Jagan Srinivasan, Omer Durak, Paul W Sternberg

**Affiliations:** 1 Howard Hughes Medical Institute, Division of Biology, California Institute of Technology, 1200 East California Boulevard, Pasadena CA – 91125, USA

## Abstract

**Background:**

Avoidance of noxious stimuli is essential for the survival of an animal in its natural habitat. Some avoidance responses require polymodal sensory neurons, which sense a range of diverse stimuli, whereas other stimuli require a unimodal sensory neuron, which senses a single stimulus. Polymodality might have evolved to help animals quickly detect and respond to diverse noxious stimuli. Nematodes inhabit diverse habitats and most nematode nervous systems are composed of a small number of neurons, despite a wide assortment in nematode sizes. Given this observation, we speculated that cellular contribution to stereotyped avoidance behaviors would also be conserved between nematode species. The ASH neuron mediates avoidance of three classes of noxious stimuli in *Caenorhabditis elegans*. Two species of parasitic nematodes also utilize the ASH neuron to avoid certain stimuli. We wanted to extend our knowledge of avoidance behaviors by comparing multiple stimuli in a set of free-living nematode species.

**Results:**

We used comparative behavioral analysis and laser microsurgery to examine three avoidance behaviors in six diverse species of free-living nematodes. We found that all species tested exhibit avoidance of chemo-, mechano- and osmosensory stimuli. In *C. elegans*, the bilaterally symmetric polymodal ASH neurons detect all three classes of repellant. We identified the putative ASH neurons in different nematode species by their anatomical positions and showed that in all six species ablation of the ASH neurons resulted in an inability to avoid noxious stimuli. However, in the nematode *Pristionchus pacificus*, the ADL neuron in addition to the ASH neuron contributed to osmosensation. In the species *Caenorhabditis *sp. 3, only the ASH neuron was required to mediate nose touch avoidance instead of three neurons in *C. elegans*. These data suggest that different species can increase or decrease the contribution of additional, non-ASH sensory neurons mediating osmosensation and mechanosensation.

**Conclusion:**

The overall conservation of ASH mediated polymodal nociception suggests that it is an ancestral evolutionarily stable feature of sensation. However, the finding that contribution from non-ASH sensory neurons mediates polymodal nociception in some nematode species suggests that even in conserved sensory behaviors, the cellular response network is dynamic over evolutionary time, perhaps shaped by adaptation of each species to its environment.

## Background

Despite a more than 1000-fold range in the sizes of different nematode species, nematode nervous systems are composed of a small number of neurons: *Caenorhabditis elegans*, which is 1 mm long, has 302 neurons [1], whereas the parasite *Ascaris lumbricoides*, which grows to be 20 cm long, has 298 neurons [2]. This constancy in number of neurons suggests a constraint in the nervous system of nematodes.

Free-living nematodes use amphids and phasmids as sensory structures to seek food and avoid harmful stimuli [3,4]. In *C. elegans*, the functions of several of the amphidial neurons and their roles in various behaviors have been characterized in detail [3-5]. Parasitic nematodes, on the other hand, use amphids to either actively seek the host (*Strongyloides stercoralis*) [6,7] or passively seek the host (*Haemonchus contortus*) [8,9]. Electron microscopic reconstruction of the amphids of the free-living nematode *C. elegans *and several parasitic nematodes such as *H. contortus *and *Ancylostoma caninum*, have shown that the sensory neuroanatomy is remarkably similar between the different species [1,6,7,10,11]. For instance, the amphid sensilla in *H. contortus*, a passively ingested parasite of sheep, shows the presence of 12 sensory neurons just like in *C. elegans *[1,8,9,11]. This conservation of neuroanatomy between *H. contortus *and *C. elegans *is in perfect correlation with the current phylogeny wherein this parasitic nematode is in the same order as *C. elegans *[12,13]. However, it is surprising that even in the distantly related nematode parasite *S. stercoralis*, there is remarkable similarity of the amphid structure with *C. elegans *[6,7,10]. Given this similarity, it is highly likely that similar cells would mediate the same behaviors across these diverse nematode species.

Studies by Schad and colleagues on the role of homologous neurons mediating chemotaxis, thermotaxis and development in parasitic nematodes have shown that positionally homologous neurons perform similar functions in these species, suggesting that neuronal function is conserved between free-living and parasitic nematodes [10,14,15]. Infective juveniles in parasitic nematodes use chemical, physical and thermal signals to find their host and resume development [14,16,17]. For instance, the finger cell AFD neurons in *H. contortus *and the dog hookworm *A. caninum *act as thermosensors just as in *C. elegans *[17,18]. Even in a more distantly related nematode *S. stercoralis*, ablation of the ASE neuron and ASH neuron resulted in loss of attraction to sodium chloride at lower concentrations and avoidance of high salt concentration respectively [19,20]. These results suggest that *C. elegans *can serve as a useful model in understanding the role of different sensory neurons in diverse nematode species.

**Figure 1:**
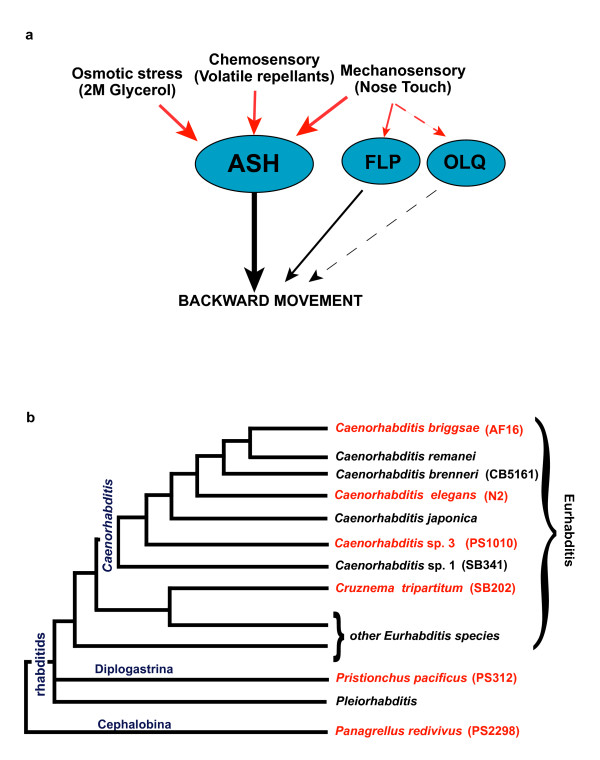
**Phylogeny of rhabditid nematode species used in our comparative analysis**. (A) Neuronal connectivity of the polymodal ASH sensory neuron modified from [27,41]. Red arrows indicate environmental stimuli perceived by the ASH neuron. The FLP and OLQ neurons play a minor role in mediating mechanosensory responses in *Caenorhabditis elegans*. Black lines indicate synaptic connections to the command interneurons that generate a backward movement in response to the stimulus. (B) Portion of the rhabditid phylogenetic tree adapted from Kiontke and Fitch [13] with species shown in red used in our analysis. Strain numbers are indicated in parentheses. The genus *Caenorhabditis *consists of different *Caenorhabditis *species including *Caenorhabditis elegans *and *Caenorhabditis briggsae *and the less closely related species *Caenorhabditis *sp. 3. *Caenorhabditis tripartitum *belongs to a different branch than the genus *Caenorhabditis *within the rhabditids. *Pristionchus pacificus *belongs to the diplogastrids and *Panagrellus redivivus *represents an outgroup to the entire group of rhabditids.

Involvement of other amphidial neurons such as the ADL neuron in chemorepulsion to SDS in *A. caninum *suggested that the sensory repertoire could change in nematodes [21]. We have now extended the work of Schad and colleagues by comparing multiple avoidance behaviors in diverse free-living nematode species to further investigate sensory response evolution. In particular, we studied avoidance responses to aversive stimuli mediated by the polymodal neuron ASH. In *C. elegans*, the bilaterally symmetric pair of 'polymodal nociceptive' ASH neurons mediates stereotyped avoidance to high osmotic strength, certain odorants, nose touch, acidic pH, heavy metals, and alkaloids [5,22-25]. Avoidance of these stimuli involves a highly stereotyped withdrawal response that is well characterized in *C. elegans *[5,26,27] (Figure 1A). The ASH neuron is also involved in mediating avoidance of high salt concentrations and the detergent sodium dodecyl sulfate (SDS) in the parasitic nematode *S. stercoralis *[20,21].

For our comparative analysis, we chose six representative free-living species from different groups of rhabditids with a range of phylogenetic distances to *C. elegans *(Figure 1B). Based on molecular data, rhabditids can be distinguished into two major clades: the Eurhabditis and the Pleiorhabditis [13]. *C. elegans*, along with other members of the *Caenorhabditis *genus, belongs to the Eurhabditis clade [13]. From the genus *Caenorhabditis*, we chose *C. elegans *(N2), *Ceanorhabditis briggsae *(AF16), and the less closely related *Caenorhabditis *sp. 3 (PS1010) [12,13]. *C. elegans *and *C. briggsae *were isolated from compost and *Caenorhabditis *sp. 3 (PS1010) was isolated from galleries of palm and sugarcane weevils [28]. *Cruznema tripartitum *(SB202) is also a member of Eurhabditis but belongs to a different branch than *Caenorhabditis *[13]. This species has been isolated from different kinds of rotting organic material, and also from garden soil and compost [29]. From the group of diplogastrids, we chose the satellite model system *Pristionchus pacificus *(PS312) [30,31], which belongs to a genus that associates with beetles [32]. *Panagrellus redivivus *(PS2298) was chosen as a representative of the outgroup and has been isolated from sugar-rich environments such as sap of rubber trees and brewery mash [33] (Figure 1B). The choice of nematode species provided a broad phylogenetic spectrum for our comparative analysis.

## Results

### Comparative neuroanatomy and identification of amphid neurons in different nematode species

Cellular anatomy is essentially invariant between individuals of the same nematode species and the number of neurons is highly conserved between nematode species, allowing the identification of neurons across different species of nematodes [6,10,17,34,35].

**Figure 2:**
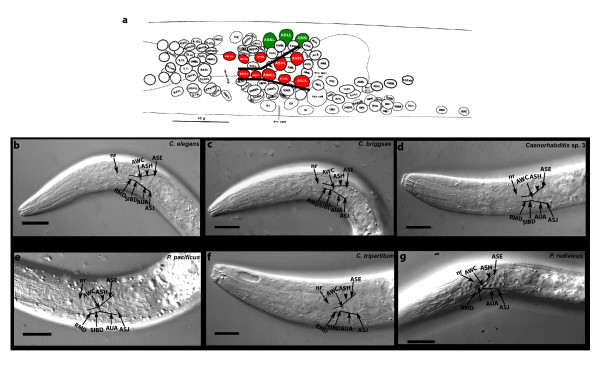
**Identification of amphidial neurons in the different nematode species**. (A) Neuronal map of the L1 larval stage the nematode *Caenorhabditis elegans *(reprinted from Sulston [35] with permission from Elsevier). The ASH neuron along with other neurons found in the same focal plane (red). The dorsal triplet amphidial neurons (green) are found in a different focal plane. The nerve ring, which surrounds the pharynx of the larva, is a process bundle with few or no cell bodies. (B-G) DIC light micrographs of the first larval stage (L1) in the different nematode species showing the amphidial cell bodies and nerve ring. B) *C. elegans *(N2), C) *Caenorhabditis briggsae *(AF16), D) *Caenorhabditis *sp. 3 (PS1010), E) *Cruznema tripartitum *(SB202), F) *Pristionchus pacificus *(PS312) and G) *Panagrellus redivivus *(PS2298). The ASH neuron (black arrowhead) is located laterally above the AUA neuron. All the neurons (ASE, ASH, AWC, AUA, ASJ) are present in the same focal plane. The SIBD and the RMD neurons posterior to the AWC neuron complete the 'Y' shaped arrangement. The nerve ring (nr) is shown (black arrow). In *C. tripartitum *and *P. redivivus*, the neurons are larger, correlating with the larger size of the nematodes. Scale bar = 50 μm.

Using the neuronal map of *C. elegans *(Figure 2A) [35], we identified the amphid cell bodies in other species. The amphidial neurons are present posterior to the nerve ring in all the species. In *C. elegans*, the ASH neuron is part of a five neuron group (ASJ, AUA, AWC, ASH, and ASE) arranged in the shape of a 'V' (Figures 2A and 2B). The position of these neurons is more lateral than all other neurons in the same focal plane. An important feature of this arrangement is the presence of two neurons anterior to the AWC neuron, RMD and SIBD (Figures 2A and 2B). These two neurons along with the five neurons form a 'Y'. We found that this arrangement is conserved in all species examined (Figures 2C–E). We observed that in all species, the position and size of the putative ASH neuron was similar to *C. elegans *(Figures 2C–E). However, in *C. tripartitum *and *P. redivivus*, the putative ASH neurons are larger than those in *C. elegans*, consistent with the larger size of these species (Figures 2F and 2G). The RMD and SIBD neurons, which are small in *C. elegans*, are also larger in these species (Figures 2F and 2G). The dorsal triplet amphid neurons ASI, ADL, and ASK are found in a different focal plane and these cells were also found in the other species in the same position (Figure 2A).

### Visualization of the amphid neuron morphology using fluorescent dye uptake in different nematode species

**Figure 3:**
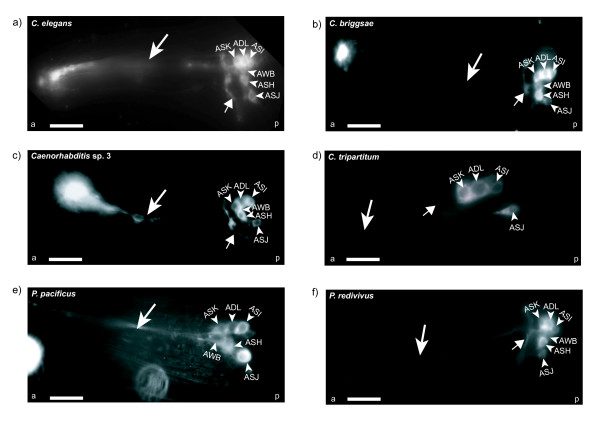
**Visualization of amphid neuron morphology in various nematodes using DiI staining**. (A-E) Morphology of the different amphid neurons was similar in most species. In *Caenorhabditis elegans*, *Caenorhabditis briggsae *and *Caenorhabditis *sp. 3, all the amphid neurons were stained brightly. *Cruznema tripartitum *did not have any uptake in the ASH and AWB neurons and *Pristionchus pacificus *also exhibited a relatively weak staining in the ASH neuron and *Panagrellus redivivus *also had weak uptake in the ASJ and AWB neurons. For each species, the arrowheads indicate the neuronal cell bodies of the different amphids. The smaller arrows indicate the nerve ring as stained by the dye. The larger arrow depicts the dendritic processes that the neurons extend to the exterior. The small letters a and p represent the anterior and posterior regions of the nematode.

In *C. elegans*, amphid and phasmid neurons take up lipophilic dyes such as DiI, DiO, and DiD from the environment [36-38]. These dyes have been used to visualize the ASI, ADL, ASK, AWB, ASH, and ASJ amphid neurons, as well as the PHA and PHB phasmid neurons [39]. We used DiI to visualize the amphid and phasmid neurons in all the species, and found that all species labeled most of the amphid neurons (Figures 3A–E). In addition, DiI staining showed that the morphology of the amphid neurons was very similar in most of the species as they label all parts of the neuron (Figures 3A–E). In *C. elegans*, all amphid neurons showed relatively strong dye uptake (Figure 3A). *C. briggsae *and *Caenorhabditis *sp. 3 also showed strong staining of all the amphid neurons (Figures 3B and 3C). In *C. tripartitum*, we could visualize only four of the six amphid neurons: ASI, ADL, ASK, and ASJ. We did not observe any staining of the ASH neuron in this species even though we were able to identify the neurons using differential interference contrast microscopy (Figures 3D and 2E). In *P. pacificus*, the ASH, ADL, and AWB neurons were stained relatively weakly compared with the other species (Figure 3E) and *P. redivivus *had the weakest staining for ASI, AWB, and ASJ neurons, respectively (Figure 3F). The differential dye uptake in the diverse species indicates that dye uptake is not sufficient to define equivalent neurons; however, it is consistent with general conserved properties of amphid neurons as having open sensory endings, and could have revealed major differences in morphology or position of neuronal cell bodies [7,11].

### ASH neuron mediates avoidance to the volatile chemical 1-octanol in all six nematode species

**Figure 4:**
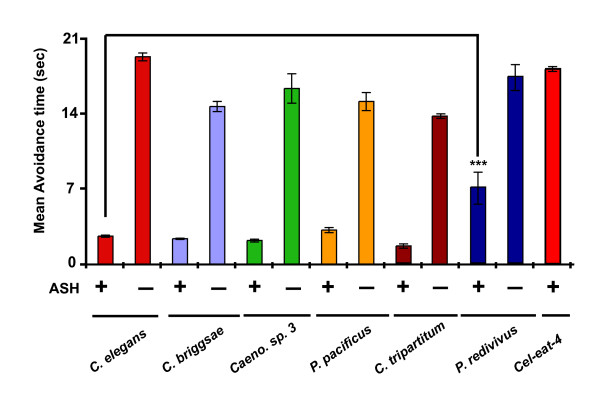
**Octanol avoidance behavior is highly conserved in nematodes and is mediated by ASH neurons**. Most species had similar avoidance times to *Caenorhabditis elegans*. *Panagrellus redivivus *showed significantly higher avoidance time to 100% octanol than *Caenorhabditis elegans*. ASH neuron ablations in different species resulted in animals not sensing 100% octanol. A mutant defective in the *C. elegans *vesicular glutamate transporter *eat-4 *was used as a negative control [56]. Data are represented as mean avoidance time (in seconds) and error bars indicate standard error of mean (s.e.m). Mean avoidance time of different species was compared by ANOVA. Presence and absence of neurons is denoted by '+' and '-' respectively. *P *values are denoted as follows: ***, *P *< 0.0001. For unablated and ablated conditions, *n *= 30 and *n *= 15 animals, respectively.

Based upon standard assays, avoidance of the volatile odorant 1-octanol is remarkably conserved across all species tested (Figure 4). *C. elegans *responds quickly to 100% octanol (~2–3 seconds) [40,41] Additonal File 1. In *C. elegans*, in the presence of food, response to 100% octanol is primarily mediated by the ASH neuron, and in the absence of food ASH, ADL, and AWB neurons are responsible for sensing 100% octanol, suggesting that feeding status of the animal affects response to 1-octanol in *C. elegans *[40]. All the species except *P. redivivus *responded after ~2–3 seconds of octanol exposure, indicating that these species are similar in sensitivity to *C. elegans *[42]. *P. redivivus *showed significantly delayed sensitivity to 1-octanol, responding in an average of 5.9 seconds. The delayed response could be due to the requirement for a higher concentration of the chemical to generate a response. To confirm the identity of the putative ASH neuron identified by position and morphology (Figures 2B–G), we used a laser microbeam to kill the ASH neuron and assayed for octanol avoidance. Ablation of the ASH neuron in all the species is sufficient to abolish 1-octanol avoidance (Figure 4). As a control, we ablated the AWC neuron in all the species and found that ablation of this neuron did not result in loss of avoidance response to 1-octanol, suggesting that the ASH neuron is specifically required for avoidance to 1-octanol in all species (Figure S1 in Additional file 2).

### Nose touch avoidance behavior shows contraction of sensory network

**Figure 5:**
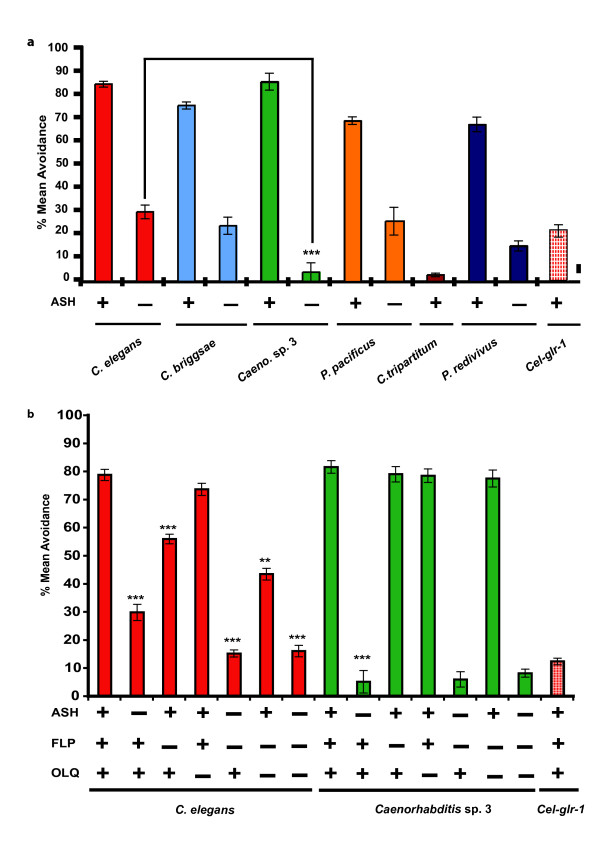
**Nose touch avoidance behavior is primarily mediated by ASH neuron in different species of nematodes**. Data are represented as mean percent avoidance and error bars indicate standard error of mean (s.e.m). (See Supplementary Materials and Methods in Additional file 1 for details). Presence and absence of neurons is denoted by '+' and '-', respectively. For unablated and ablated conditions, *n *= 30 and *n *= 10 animals, respectively. (A) Most species exhibit similar avoidance responses when challenged with a mechanosensory stimulus. Ablation of the ASH neurons results in reduction of nose touch avoidance in all species. In *Caenorhabditis *sp. 3 (PS1010), ASH ablation results in complete abolishment of nose touch response compared with ASH ablated animals in *Caenorhabditis elegans*. Mean percent avoidance of different species was compared using ANOVA. *P *values are denoted as follows: ***, *P *< 0.001. (B) FLP and OLQ neurons do not mediate nose touch response in *Caenorhabditis *sp. 3. In *C. elegans*, FLP-ablated animals were significantly different than unablated animals. ASH/FLP-ablated animals were significantly different than ASH-ablated animals. OLQ ablations did not affect nose touch in *C. elegans*. Ablation of FLP/OLQ had significantly lower nose touch response than either of the single ablated animals. Ablations of ASH/FLP/OLQ-ablated animals had significantly lower response than ASH-ablated animals but were similar in response to ASH/FLP-ablated animals in *C. elegans*. In *Caenorhabditis *sp. 3, FLP-ablated animals were not significantly different than unablated animals (*P *> 0.05). There were no significant differences in nose touch avoidance between ASH-ablated, FLP/ASH-ablated, and ASH/FLP/OLQ-ablated animals (*P *> 0.05). Other ablations tested for statistical significance: ASH-ablated vs. unablated animals, *P *< 0.001; FLP-ablated vs. ASH/FLP-ablated, *P *< 0.001; FLP-ablated vs. FLP/OLQ-ablated, *P *< 0.01; OLQ-ablated vs. FLP/OLQ-ablated, *P *< 0.001. *P *values were generated by ANOVA and denoted as follows: **, *P *< 0.01; ***, *P *< 0.001.

Nose touch avoidance behavior also showed a high degree of conservation among five of six species tested (Figure 5A). In *C. tripartitum*, after the first few positive trials, the animals failed to respond to nose touch, showing an overall avoidance of < 10%, suggesting that *C. tripartitum *adapts to the nose touch stimulus faster than *C. elegans *and the other species tested (Figure 5A). However, during the first three trials, *C. tripartitum *showed a quantitatively similar response to that of *C. elegans *(Figure S2 in Additional file 2). Ablation of the ASH neurons in five nose-touch responsive species resulted in loss of nose touch avoidance (Figure 5A). Four of five species showed nose touch responses in 20–30% of the trials (Figure 5A). However, in *Caenorhabditis *sp. 3, ASH-ablated animals completely lacked the ability to respond to nose touch (Figure 5A). Ablation of the AWC neuron did not affect response to nose touch in the different species (Figure S3 in Additional file 2).

In *C. elegans*, nose touch response is mediated by two non-amphid neurons, FLP and OLQ, in addition to the ASH neurons [25]. To test whether nose touch response might be mediated by a single sensory neuron (ASH) and to check the role of the non-amphid neurons, we assayed nose touch response in FLP-ablated, OLQ-ablated, FLP/OLQ-ablated and FLP/OLQ/ASH-ablated animals in both *C. elegans *and *Caenorhabditis *sp. 3 (Figure 5B). In *C. elegans*, FLP-ablated animals were significantly less responsive to nose touch compared with unablated animals. OLQ-ablated *C. elegans *showed no effect on nose touch compared with unablated animals (Figure 5B). However, ablation of both the non-amphid neurons FLP and OLQ in *C. elegans *resulted in significantly decreased nose touch avoidance compared with either of the single neuron ablations, suggesting that among the non-amphid neurons, FLP plays a major role in mediating nose touch whereas OLQ plays a minor role [25] (Figure 5B). In *C. elegans*, FLP/ASH-ablated animals were significantly different from ASH-ablated animals (Figure 5B). Animals lacking FLP, ASH, and OLQ neurons were significantly different from ASH-ablated animals but not different from ASH/FLP-ablated animals (Figure 5B).

In *Caenorhabditis *sp. 3, the FLP and OLQ neurons have no effect on nose touch response (Figure 5B). Moreover, ablation of both the two non-amphid neurons (FLP, OLQ) did not show any significant difference from either of the single ablated animals (Figure 5B). In *Caenorhabditis *sp. 3, ablation of all three neurons (ASH, FLP, and OLQ) did not significantly differ from ASH-ablated animals (Figure 5B). These results suggest that the ASH neuron completely mediates nose touch avoidance behavior in *Caenorhabditis *sp. 3, as compared with three neurons in *C. elegans*. It is conceivable that in *Caenorhabditis *sp. 3, other neurons could be involved in mediating nose touch behavior. However, ASH-ablated animals in *Caenorhabditis *sp. 3 show less of a nose touch response compared with ASH/FLP/OLQ-ablated animals in *C. elegans*, suggesting that ASH mediates most of the nose touch response in *Caenorhabditis *sp. 3 (Figure 5B). The sensory response network for mechanical stimuli in *Caenorhabditis *sp. 3 thus shows a reduction in the number of neurons involved compared with *C. elegans*.

### Expansion of the sensory network mediating osmotic avoidance behavior

**Figure 6:**
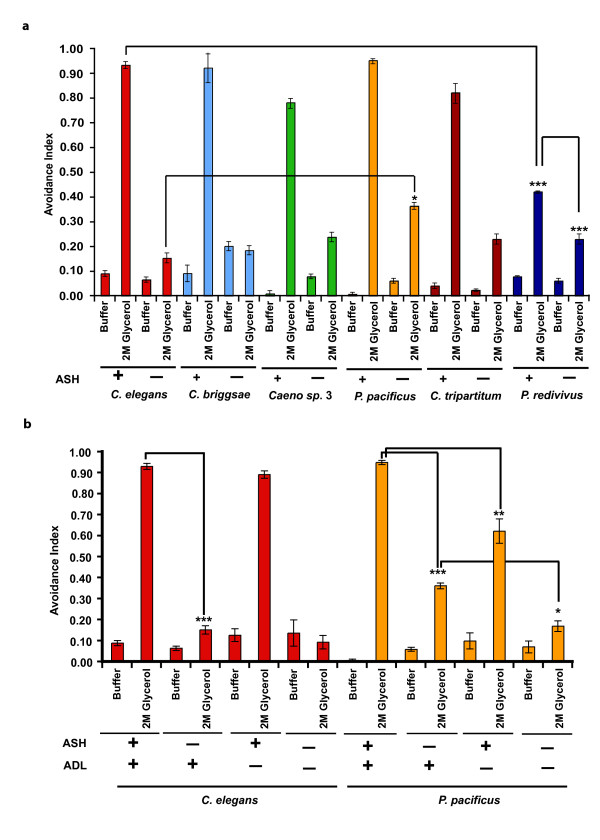
**Osmotic avoidance behavior evolves at the cellular level in different nematodes**. Data are represented as mean avoidance index (see Supplementary Materials and Methods in Additional file 1 for details) and error bars indicate standard error of mean (s.e.m). Presence and absence of neurons is denoted by '+' and '-', respectively. Ablated animals were tested with 2 M glycerol. For unablated and ablated conditions, *n *= 30 and *n *= 10 animals, respectively. (A) Most species showed similar responses to osmotic avoidance to *Caenorhabditis elegans*. *Panagrellus redivivus *(PS2298) showed significantly low sensitivity to both 1 M and 2 M glycerol concentrations compared with *C. elegans*. In all species tested, ablation of the ASH neuron resulted in failure of animals to avoid osmotic stress. Ablation of ASH neurons in *P. redivivus *resulted in animals not responding to the 2 M glycerol. ASH-ablated *C. elegans *significantly differ from ASH-ablated *Pristionchus pacificus*. *P *values were generated by ANOVA for the different species and are denoted as follows: *, *P *< 0.05; ***, *P *< 0.0001. (B) Cellular additivity of osmotic avoidance behaviors in *P. pacificus*. Ablation of ADL neuron had no effect on osmotic avoidance in *C. elegans*. ADL-ablated animals in *P. pacificus *had a significantly lower avoidance index than unablated animals. Ablation of ASH and ADL neurons in *P. pacificus *resulted in complete loss of osmotic avoidance. *P *values were generated using ANOVA. *P *values are denoted as follows: *, *P *< 0.05; **, *P *< 0.01; ***, *P *< 0.001.

Osmotic avoidance behavior was assayed using the standard drop assay [23] (Figure 6A). *C. elegans *responds within a second of drop delivery by backing away from the drop of osmotic solution [23]. The avoidance index (a.i.) was calculated by dividing the number of positive responses to the total number of trials [23] (Additional file 1). Within the *Caenorhabditis *clade, all three species tested responded similarly to *C. elegans *with respect to response times to 2 M glycerol. However, the phylogenetically distant species, *P. pacificus*, *C. tripartitum*, and *P. redivivus*, had longer response times compared with *C. elegans *(Table 1). The average response times for *P. pacificus, C. tripartitum*, and *P. redivivus *were 5.2, 7.4, and 8.1 seconds, respectively (Table 1). *P. redivivus*, the most distant species tested, showed significantly decreased sensitivity to 2 M glycerol compared with the other species (Figure 6A). We speculate that *P. redivivus *could be adapted to high osmotic conditions because it was initially isolated from high sugar concentration environments.

**Table 1:** Comparison of mean response times to 2 M glycerol in free-living nematodes

	**Species**	**Mean response time (s)**
**1**	*Caenorhabditis elegans*	1.1
**2**	*Pristionchus pacificus*	5.2
**3**	*Cruznema tripartitum*	7.4
**4**	*Panagrellus redivivus*	8.1

In *Caenorhabditis elegans*, the animal backs away from the stimulus almost instantaneously within a second. However, avoidance to high osmolarity conditions by more distant species varies. *Panagrellus redivivus *was the slowest in response time in our assay. Data represented here is the average response time of 40 animals per species tested over multiple days.

Ablation of the ASH neuron almost completely abolishes osmotic avoidance response in *C. elegans *[23]. In the parasitic nematode *S. stercoralis*, avoidance response to high salt concentrations is completely mediated by the ASH neuron [20]. Ablation of the ASH neuron in the other species resulted in reduced a.i. (Figure 6A). ASH ablation does not completely abolish osmotic avoidance in *P. pacificus*, suggesting the possibility of ASH-independent mechanisms of osmotic avoidance (*C. elegans *a.i. = 0.15 vs. *P. pacificus *a.i. = 0.36; Figure 6A). In *C. elegans*, ASH, ADL, ASK, and ASE neurons are known to mediate avoidance of chemical repellents [23]. The residual osmotic avoidance seen in ASH ablated animals of *P. pacificus *could be mediated by these neurons. In *C. elegans*, ablation of the ADL neuron had no effect on osmotic avoidance (Figure 6B). Ablation of both ASH and ADL neurons in *C. elegans *did not show a significant change in osmotic avoidance index compared with ASH-ablated animals (Figure 6B). In *P. pacificus*, ablation of ADL neuron results in a significantly lower avoidance index compared with unablated animals (Figure 6B). Ablation of both ASH and ADL neurons in *P. pacificus *had a significantly lower avoidance index compared with ASH-ablated or ADL-ablated animals, suggesting an additive effect of both the neurons in osmotic avoidance behavior (ASH/ADL-ablated a.i. = 0.17 vs. ASH-ablated a.i. = 0.36; ASH/ADL-ablated a.i. = 0.17 vs. ADL-ablated a.i. = 0.62) (Figure 6B). Ablation of the ASK and ASE neurons did not change the osmotic sensitivity of *P. pacificus*, indicating that these neurons do not play any role in osmotic avoidance (data not shown). Hence, we believe that the ASH neuron is the primary sensory neuron for mediating osmotic avoidance in all nematode species, but additional sensory neurons, such as ADL neuron in *P. pacificus*, may contribute to mediating osmotic avoidance.

In *C. elegans*, the ADL neuron, along with ASH and ASE neurons, mediates avoidance response to heavy metal ions, but does not play a role on its own in mediating this avoidance [43]. In the dog hookworm *A. caninum*, both ASH and ADL neurons are required to mediate avoidance of the detergent sodium dodecyl sulphate [21]. Ablation of only the ADL neurons in the hookworm results in an assortment of responses, from avoidance to non-responsive behavior, suggesting that the animal is not able to decide whether to respond to the chemical [21]. In *P. pacificus*, ablation of ADL neuron resulted in a significant reduction of avoidance response, indicating that this neuron is necessary for mediating avoidance to high osmotic conditions. The role of the ADL neuron during avoidance behaviors in different species thus remains unclear. Perhaps analysis of the genes expressed in the ADL neuron would provide insight into the role of this neuron in the different species.

We cannot rule out the possibility that ablation of specific neurons results in functional replacements by other neurons. However, if this occurs, then this aspect of nematode neurobiology varies among cells, behaviors, and species.

### Sensory sensitivity to noxious stimuli varies between different nematode species

We hypothesized that nematodes would display habitat-specific sensitivity to the same stimuli rather than phylogeny based sensitivity. Therefore, we tested several different concentrations of the different noxious stimuli (chemosensation and osmosensation), and used a cluster algorithm to generate a behavioral dendrogram for the different avoidance behaviors (see Methods).

**Figure 7:**
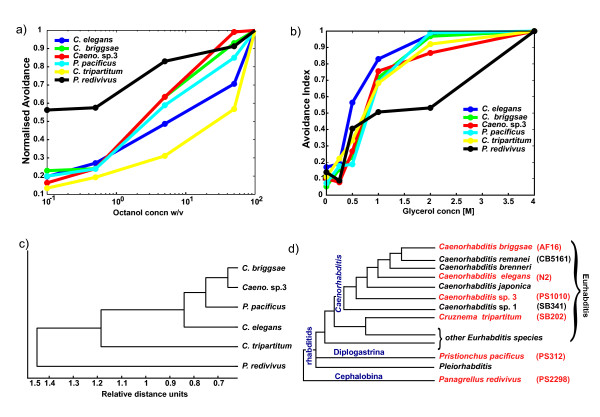
**Sensory sensitivity to different concentrations of the stimuli varies in the different nematode species**. (A) Concentration curves for octanol avoidance. We tested concentrations ranging from 0% octanol to 100% octanol on the species and found that *Panagrellus redivivus *did not avoid this noxious chemical to the same extent as the other species. *Caenorhabditis elegans *and *Cruznema tripartitum *displayed similar trends in avoidance and *Caenorhabditis briggsae, Caenorhabditis *sp. 3 and *Pristionchus pacificus *responded very similarly. Data for each species is represented as normalized avoidance index (see Methods for details). (B) Concentration curves for glycerol avoidance. Concentrations ranging from 0.1 M to 4 M glycerol were tested on the different nematodes. We found that *P. redivivus *showed least avoidance of glycerol at concentrations wherein the other species fully responded to glycerol. At the highest concentration tested, *P. redivivus *also responded strongly to glycerol. All the other species displayed similar avoidance of different concentrations of glycerol. Data for each species is represented as normalized avoidance index (See Methods for details). (C) Behavioral dendrogram for osmotic avoidance and octanol avoidance and its relation to the phylogenetic tree. The dendrogram was generated using hierarchical clustering for these two sensory behaviors. This data set incorporated all the different ablations done in the different species for the different behavioral assays. As expected *P. redivivus *and *C. tripartitum *exhibited the most different behavioral repertoire compared with the other species. (D) Phylogenetic tree of the species used in our analysis.

For octanol avoidance, we tested concentrations ranging from 0.1% to 100% octanol. The data for the different species was then normalized for each concentration with the maximal value for that concentration giving a normalized avoidance index for the different species (see Methods). *P. redivivus *displayed the least sensitivity to octanol compared with the other species at the different concentrations (Figure 7A). *C. elegans *and *C. tripartitum *show similar sensitivity to 1-octanol (Figure 7A). Also, in *C. briggsae*, *Caenorhabditis *sp. 3, and *P. pacificus*, sensitivity to octanol avoidance was more similar to each others' than to the other species' (Figure 7A).

As with osmotic avoidance behavior, we found that *P. redivivus *was the least sensitive to glycerol compared with the other species (Figure 7B). This observation correlates with the fact that *P. redivivus *has been isolated from high osmotic strength environments and hence could be adapted to high osmolarity [33,44]. However, at the highest concentration tested (4 M glycerol), *P. redivivus *also exhibited a very high avoidance, as did the other species (Figure 7B). *C. briggsae, Caenorhabditis *sp. 3, *C. tripartitum*, and *P. pacificus *exhibited similar sensitivity to osmotic avoidance (Figure 7B). *C. elegans *exhibited slightly different osmotic sensitivity different from all these species with the exception of *P. redivivus *(Figure 7B). Hence, other than *P. redivivus*, all other species exhibited similar sensitivity to different osmolarity conditions.

Finally, we combined the data of octanol and osmotic avoidance behaviors including the ASH neuron ablation data for all the species and found that *P. redivivus *exhibited the most different behavior in our analyses (Figure 7C). Based on relative distances computed by the algorithm, *C. tripartitum *is the next closely related species to *P. redivivus*. *C. elegans*, along with the other species, forms a whole branch on the behavioral dendrogram, with *P. pacificus *being closely related to it. *C. briggsae *and *Caenorhabditis *sp. 3 form a sub-branch, suggesting that these two exhibit similar behaviors. Comparing our behavioral dendrogram for octanol and osmotic avoidance behaviors with the phylogenetic tree, we see some interesting features (Figures 7C and 7D). We observe that for these two behaviors, the relative positions of *P. redivivus *and *C. tripartitum *resemble that of the phylogenetic tree (Figures 7C and 7D). *P. pacificus *seems to be behaviorally similar to the branch of *Caenorhabditis *sp. 3 and *C. briggsae*. Given the association of *Caenorhabditis *sp. 3 with rice weevils [28] and *P. pacificus *with beetles [45], this correlation makes sense. Unexpectedly, *C. elegans *does not exhibit similar behavioral properties like its close siblings *C. briggsae *and *Caenorhabditis *sp. 3. These data suggest that sensitivity to different stimuli varies among species and that the differential sensitivity could be linked to the functional sensory receptor repertoire of these species [46].

## Conclusion

By comparing multiple aversive behaviors in many nematode species at a cellular level, we demonstrate the relative flexibility of the sensory system of nematodes. This suggests that the sensory architecture mediating certain polymodal behaviors can evolve (Table 2). We observed that all species tested in our analysis avoided the three different aversive stimuli with the exception of the nematode *C. tripartitum*, which showed a high degree of adaptation to nose touch. Similarly, parasitic nematodes also avoided several aversive stimuli [14,18,34,47]. Such conservation of avoidance behaviors in the different nematode species suggests that natural selection maintains these behaviors [48,49].

**Table 2:** Comparison of sensory response networks in free-living nematodes

**Stimulus**	**Contributing cells**	**Species**		
		***Caenorhabditis elegans***	***Caenorhabditis *sp. 3**	***Pristionchus pacificus***
Octanol	ASH	+++	+++	+++
Nose touch	ASH	++	+++	++
	FLP	+	--	ND
	OLQ	(+)	--	ND
				
Osmotic	ASH	+++	+++	++
stress	ADL	--	--	+

Most species respond similarly to the volatile odorant 1-octanol. This response is completely mediated by the ASH neuron. However, nose touch and osmotic avoidance responses evolve: in *Caenorhabditis elegans*, three neurons mediate nose touch whereas in *Caenorhabditis *sp. 3, only ASH mediates this response. Avoidance to high osmolarity conditions is primarily mediated by the ASH neuron, but in *Pristionchus pacificus*, the ADL neuron also contributes to this sensory response. '+' denotes the degree of involvement of the neuron in mediating the behavior; '+++', completely mediates; '++', partially mediates; '+', weakly mediates; '(+)', mediates but only observable when other neurons are ablated; '-', not involved; ND, not tested.

At a cellular level, avoidance response to the chemical 1-octanol was mediated by the ASH neuron in all species. In parasitic nematodes, avoidance of high salt concentration and sodium dodecylsulfate is also mediated by the ASH neuron [20,21]. However, for nose touch response behavior, we observed a reduction in the number of sensory neurons relative to *C. elegans*, with only the ASH neuron mediating this response in *Caenorhabditis *sp. 3 as compared with three neurons in *C. elegans *(ASH, FLP, and OLQ). On the other hand, we see an increase in the set of sensory neurons mediating osmotic stress with the ADL neuron partially mediating osmotic avoidance in *P. pacificus *along with the ASH neuron (Table 2). We also observed that sensory sensitivity to certain stimuli varied between the different species tested. These differential responses could be attributed to adaptation of the species to their respective niches. For example, *P. redivivus *has a slower response time in response to 1-octanol and is highly resistant to high osmotic conditions (Figures 4 and 6). Since *P. redivivus *was isolated from the sap of rubber trees and brewery mash, these responses could be attributed to adaptation to sugar-rich environments.

The conservation of several properties of a multi-functional neuron across diverse lifestyles is not surprising, given the constancy of neuron number in these nematodes. However, to understand how sensory responses evolve, comparison of closely related species is an essential prerequisite. Such studies can uncover selection pressures that act within a shared evolutionary history to cause either differences in their behavior or changes in cellular contribution to these behaviors [50]. These changes may evolve by alterations in the number and type of sensory receptors each neuron expresses, by the efficacy of signal transduction in sensory neurons, or by the strength of connectivity of the neuron to its postsynaptic partners. Our results provide a clear demonstration of change in the relative contributions of nociceptive neurons in a sensory network in diverse free-living nematode species. These studies allow inference of ways in which sensory responses in free-living nematodes might evolve, and suggest how such evolution might occur in medically and agriculturally relevant nematodes.

## Methods

### Nematode cultures and maintenance

All strains were raised at 20°C unless indicated otherwise, using standard methods [51]. Strains used in this study were *C. elegans *(N2 Bristol isolate) [51], *C. elegans eat-4*(*ky5*) MT6308 [52], *C. elegans glr-1*(*n2461*) KP4 [41], *C. briggsae *(AF16)[53], *Caenorhabditis *sp. 3 (PS1010), *P. pacificus *(PS312) [54], *C. tripartitum *(SB202), and *P. redivivus *(PS2298/MT8872) [55].

### Staining of amphid neurons using the lipophilic dye DiI

A stock solution of DiI (2 mg/ml) was prepared in dimethyl formamide and stored at -20°C [38]. Worms from the different species were washed from plates and resuspended in a small eppendorf tube to a 100 μl volume. The worm pellet was washed thrice in M9 buffer and worms allowed to settle down using gravity. To this pellet we added a 1:200 dilution of DiI solution and incubated in the dark for 2–3 hours. After the incubation, the worms were washed three times in M9 buffer and then mounted on a slide to visualize the amphid neurons. For some species, we used a longer incubation time (5–7 hours) to visualize neurons.

### Laser ablations and behavioral assays

For all species tested, we used the L1 larva stage for our ablations as described previously [26]. All behavioral assays were performed as described previously [23,25,42]. A detailed description of the ablations and behavioral assays is given in the Supplementary Materials and Methods in Additional file 1. Ablation of one of the two bilaterally symmetrical neurons did not affect the avoidance response to different stimuli.

### Sensory sensitivity assays and cluster analysis of the behavioral phenotypes

For octanol avoidance sensitivity, we made different dilutions of 100% octanol (0.1%–100%) and each species was tested with each concentration at least on three different days. Data was represented as average avoidance time for each concentration.

Similarly, for sensitivity to osmotic avoidance, we tested concentrations ranging from 0.1 M to 4 M glycerol. Data was represented as mean a.i. (see Supplementary Materials and Methods in Additional file 1 for details).

For each of the behavioral assays, the normalized a.i. was computed for each species. This was done by normalizing the value for each species at each concentration to the maximum value for that concentration. The normalized values for the different concentrations were then plotted for each species in Matlab. For obtaining the dendrogram, data was then clustered using a Matlab hierarchical clustering algorithm (Euclidean distances and average linkage). The x-axis of the dendrogram indicates relative distance between the different species.

### Statistical analysis

The statistical tests employed are described in Additional file 1.

## Authors' contributions

JS and PWS designed the experiments, JS and OD performed the experiments, and JS wrote the paper with input from PWS.

## Supplementary Material

Additional file 1 **Supplementary Materials and Methods**. Supplemental data including detailed Materials and Methods and Additional figure legends. Click here for file

Additional file 2 **Additional Figures S1–4**. Additional figures. Click here for file
