# Supplementary material for: Evolution of a polymodal sensory response network
Source: BMC Biol. 2008 Dec 15;6:52. doi: 10.1186/1741-7007-6-52 (PMC2636771; doi:10.1186/1741-7007-6-52)
Supplement: Additional file 1 — Supplementary Materials and Methods. Supplemental data including detailed Materials and Methods and Additional figure legends. [file 1741-7007-6-52-S1.pdf]

Supplementary Information

Supplemental Materials and Methods

Behavioral assays:

a) Octanol Avoidance Assays: Octanol (1-octanol) avoidance was assessed by the "smell-on-a-stick" assay as described [2]. 100% octanol was used in our assays as response to this concentration was mediated solely by the ASH neurons [3]. The blunt end of an eyelash hair was pasted to a Pasteur pipette and dipped in octanol. This hair was then placed in front of a forward moving animal's nose, and the time required by the animal to initiate backward movement was determined using an audible timer [3]. Octanol avoidance assays were conducted on well-fed worms on NGM plates containing no food as feeding status affects the response of animals to octanol [3]. For each of the species tested, we used *Caenorhabditis elegans* controls *viz*. N2 and *eat-4* to check that the assays were working properly. N2 reversed within 3 seconds of exposure, whereas *eat-4* does not respond at all [4]. Each species was tested on multiple days (n≥ 4days) and on each day at least 5-10 animals were tested.

b) Nose Touch Avoidance Assays: Nose touch avoidance was assayed by placing a hair on the surface of the plate in front of the animal perpendicular to the direction of movement as previously described [5]. These assays were conducted on NGM plates containing a thin layer of bacterial lawn. This was done by spreading 100 μl of an overnight culture of *E. coli* OP50 and allowing it to dry for a couple of hours. Avoidance behavior was quantified as the percentage of trials in which the animals responded to touch with an eyelash by stopping forward motion or initiating a reversal. No more than 10 trials were conducted on a single worm at a time and a minimum of 10 animals was tested for each species and genotype. Each species was tested on at least 5 different days to ensure data validity. For each of the species tested, we used N2 and *Cel- glr-1* was used as positive and negative controls for nose touch assays [1].

c) Osmotic avoidance assays: The drop assay was used to test the behavior of animals to osmotic stress as previously described [6]. A drop of solution containing the osmotic solution (2M glycerol) or the buffer was delivered near the tail of a moving animal. The drop surrounds the animal and reaches the anterior amphid sensory neurons. Animals responding to the osmotic stress usually initiate a backward motion away from the drop. Such a response was scored as a positive response. Drop tests were conducted on unseeded NGM plates using adult animals. The avoidance index (a.i.) for either population or single worm assays was calculated by dividing the number of positive responses to the total number of trials [6]. All species were tested using population assays. For population assays, 50 individual animals per species were tested on each day, with freshly prepared 1M and 2M glycerol concentrations to check for sensitivity to different osmotic strengths. For each species tested, *Caenorhabditis elegans* (N2) was used as a control on the same day to check for any discrepancies in solutions. Assays were conducted on multiple days, n≥ 4 days and the resulting mean represented as mean

avoidance index. All ablated animals were tested individually with 2M glycerol. An interstimuli interval (ISI) of 2 minutes is used between successive trials to the same animal. No more than 20 drops per set were carried out and not more than 3 sets were done on the same animal per day.

Laser ablations:
For all species tested, we used the L1 larva stage for our ablations. 10 mM sodium azide was used as an anesthetic for most of the species, except *Cruznema tripartitum* (SB202) and *P. redivivus* (PS2298), for which 2mM levamisole was used. Laser ablations were performed as described previously [1]. Animals were allowed to recover at 20°C for 3 days on plates containing food. On the day of the assay, the animals were transferred from 20°C to room temperature for at least an hour before conducting the assay. Individual experimental animals together with unoperated animals were assayed as young adults at room temperature (24°C-25°C), 48 hours post L4 stage by the experimenter blind to the operative status.

Statistical Analysis:
Since all the assays were done on different days, we tested for any changes in the value of means between different days and found no effect between assays run on different days, when we assayed nose touch and octanol avoidance in any nematode species (ANOVA: nose touch avoidance (P= 0.322) and mean avoidance time (P= 0.137). Therefore, we pooled results from different days for further statistical analysis.

Figure 4: For octanol avoidance, mean avoidance time of different species was compared by ANOVA. The variable factors included nematode species and the ablation status respectively. All species tested responded like *Caenorhabditis elegans* (ANOVA: Species (P<0.0001) and Ablation status (P<0.0001). Tukey HSD post hoc multiple comparison tests for octanol avoidance showed that *Caenorhabditis elegans*, *Caenorhabditis* sp. 3 and *P. pacificus* responded similarly, whereas *C. briggsae* and *C. tripartitum* responded similarly and *P. redivivus* showed different response time compared to other species. P values are denoted as follows: ***, P < 0.0001.

Figure 5A: Nose touch avoidance was represented as percent (%) mean avoidance in our assays. All species tested responded like *Caenorhabditis elegans* and ablation of ASH resulted in loss of nose touch response (ANOVA: Species (P<0.0001) and Ablation status (P<0.0001). Tukey HSD post hoc multiple correction tests showed that *Caenorhabditis elegans*, *P. pacificus* and *P. redivivus* showed different avoidance responses, whereas *C. briggsae* and *Caenorhabditis sp.* 3 showed similar responses to nose touch avoidance and fall into one group.

Figure 5B: Unablated and ablated animals of *Caenorhabditis elegans* and *Caenorhabditis* sp. 3 were compared against each other. P values were generated by ANOVA using the Tukey-Kramer Multiple Comparison test. P values are denoted as follows: *, P < 0.05; **, P < 0.01; ***, P < 0.001.

Figure 6A: Statistical analyses of osmotic avoidance between different species was calculated using 2-factor ANOVA. We performed the Tukey–Kramer Multiple Comparisons test to check the significance between ASH-ablated *Caenorhabditis elegans* and *P. pacificus*. Comparison of unablated and ablated animals between the same species was determined by the unpaired t-test with Welch correction using the InStat statistics software. *P* values are denoted as follows: **, $P < 0.01$; ***, $P < 0.0001$.

Figure 6B: We used 1-factor ANOVA for comparing the significance between the various cellular ablations. *P* values were generated using the Tukey-Kramer Multiple comparisons post hoc test. *P* values are denoted as follows: *, $P < 0.05$; **, $P < 0.01$; ***, $P < 0.001$.

Supplementary Figure S1: The AWC ablated and unablated animals for 1-octanol avoidance for each species were compared using the unpaired t-test with Welch's correction.
Supplementary Figure S2:*Caenorhabditis elegans* and *C. tripartitum* were compared using the unpaired t-test with Welch's correction.

Supplementary Figure S3-S4: The AWC ablated and unablated animals for each species were compared using the unpaired t-test with Welch's correction.

Supplementary Figure Legends:

Figure S1: **Octanol avoidance behavior in different species is not mediated by the AWC neuron.**
Data is represented as mean avoidance time (in seconds) and error bars indicate s.e.m. Presence and absence of AWC neurons is denoted by '+' and '–' respectively. Mean avoidance time for the ablated animals was compared to the unablated animals by unpaired t-test with Welch's correction (P= 0.1, unpaired t-test). For unablated and ablated conditions, $n \geq 10$ and $n \geq 10$ animals, respectively.

Figure S2: Nose touch avoidance of first three trials of *C. tripartitum* is similar to that of *Caenorhabditis elegans*. Quantification of mean nose touch avoidance indicated that there was no difference between *Caenorhabditis elegans* and *C. tripartitum* (P= 0.1, unpaired t-test).

Figure S3: AWC neurons do not play a role in nose touch avoidance behavior in the different nematodes.
Data are represented as mean percent avoidance and error bars indicate s.e.m. Presence and absence of the AWC neurons is denoted by '+' and '–', respectively. Ablation of the AWC neurons did not result in loss of nose touch behavior (unpaired t-test with Welch's correction). For unablated and ablated conditions, $n \geq 10$ and $n \geq 10$ animals,

respectively. Since *C. tripartitum* did not show significant nose touch behavior, we did not ablate the AWC neurons in that species.

Figure S4: Osmotic avoidance in different nematodes is unaffected by loss of AWC neurons.

Data are represented as mean avoidance index and error bars indicate standard error of mean (s.e.m). Presence and absence of AWC neurons is denoted by '+' and '–', respectively. 2M glycerol was used to test both ablated and unablated animals in the different species. Comparison of avoidance index of ablated animals with unablated animals was computed using unpaired t-test). For unablated and ablated conditions, n ≥ 10 and n ≥ 10 animals, respectively.

References:

1. Hart AC, Kass J, Shapiro JE, Kaplan JM: **Distinct signaling pathways mediate touch and osmosensory responses in a polymodal sensory neuron**. *J Neurosci* 1999, **19**(6):1952-1958.
2. Troemel ER, Chou JH, Dwyer ND, Colbert HA, Bargmann CI: **Divergent seven transmembrane receptors are candidate chemosensory receptors in *Caenorhabditis elegans*.** *Cell* 1995, **83**(2): 207-18.
3. Chao MY, Komatsu H, Fukuto HS, Dionne HM, Hart AC: **Feeding status and serotonin rapidly and reversibly modulate a *Caenorhabditis elegans* chemosensory circuit**. *Proc Natl Acad Sci U S A* 2004, **101**(43):15512-15517.
4. Hart AC, Sims S, Kaplan JM: **Synaptic code for sensory modalities revealed by *Caenorhabditis elegans* GLR-1 glutamate receptor**. *Nature* 1995, **378**(6552):82-85.
5. Kaplan JM, Horvitz HR: **A dual mechanosensory and chemosensory neuron in *Caenorhabditis elegans***. *Proc Natl Acad Sci U S A* 1993, **90**(6):2227-2231.
6. Hilliard MA, Bargmann CI, Bazzicalupo P: ***Caenorhabditis elegans* responds to chemical repellents by integrating sensory inputs from the head and the tail**. *Curr Biol* 2002, **12**(9):730-734.
