# Supplementary material for: Evolution of a polymodal sensory response network
Source: BMC Biol. 2008 Dec 15;6:52. doi: 10.1186/1741-7007-6-52 (PMC2636771; doi:10.1186/1741-7007-6-52)
Supplement: Additional file 2 — Additional Figures S1–4. Additional figures. [file 1741-7007-6-52-S2.pdf]

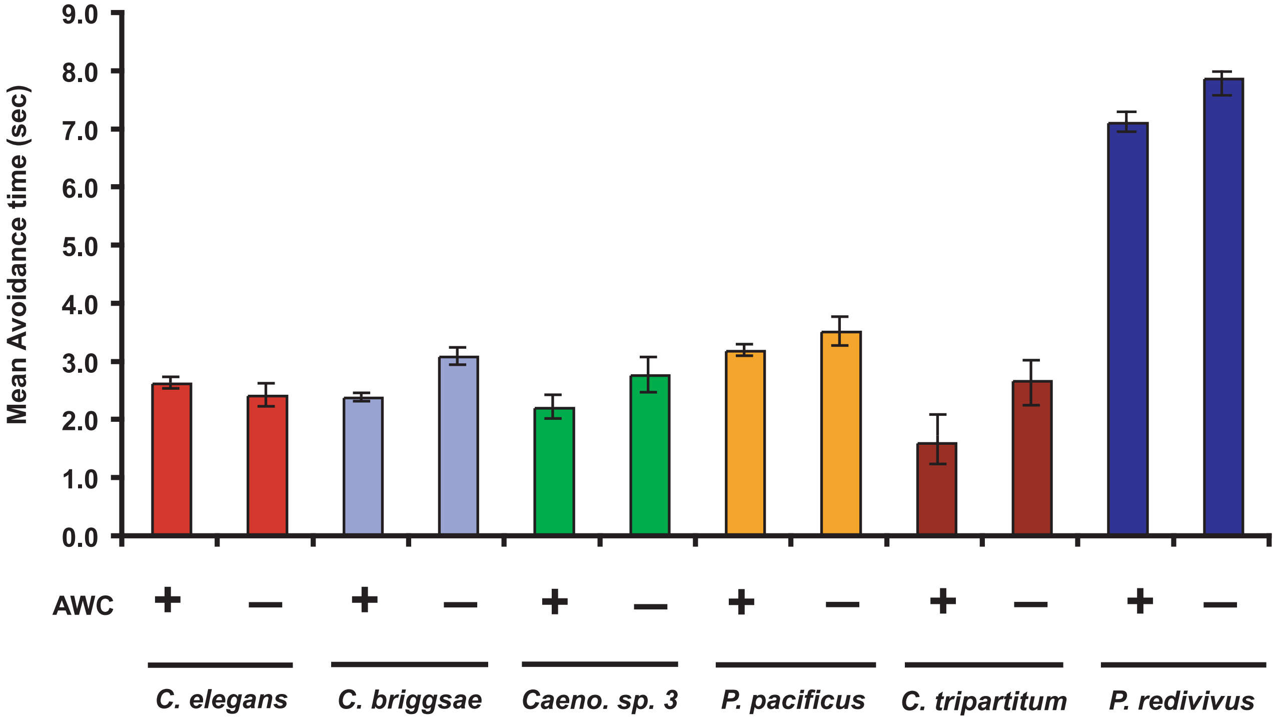

Supplementary Figure S1

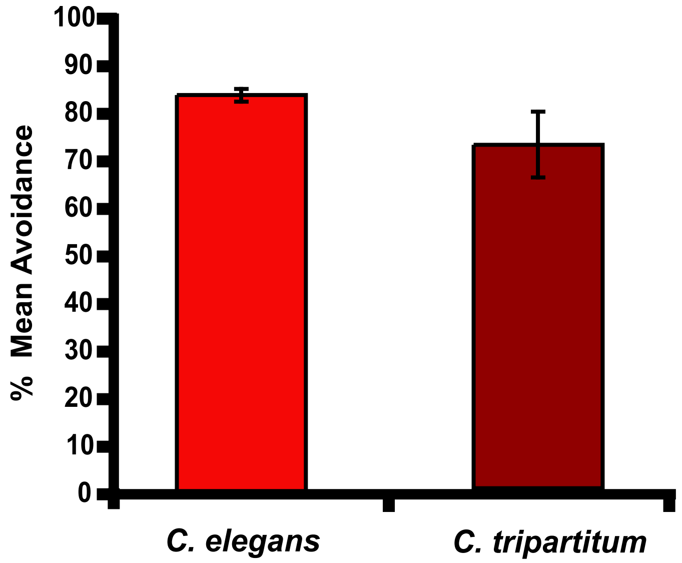

Supplementary Figure S2

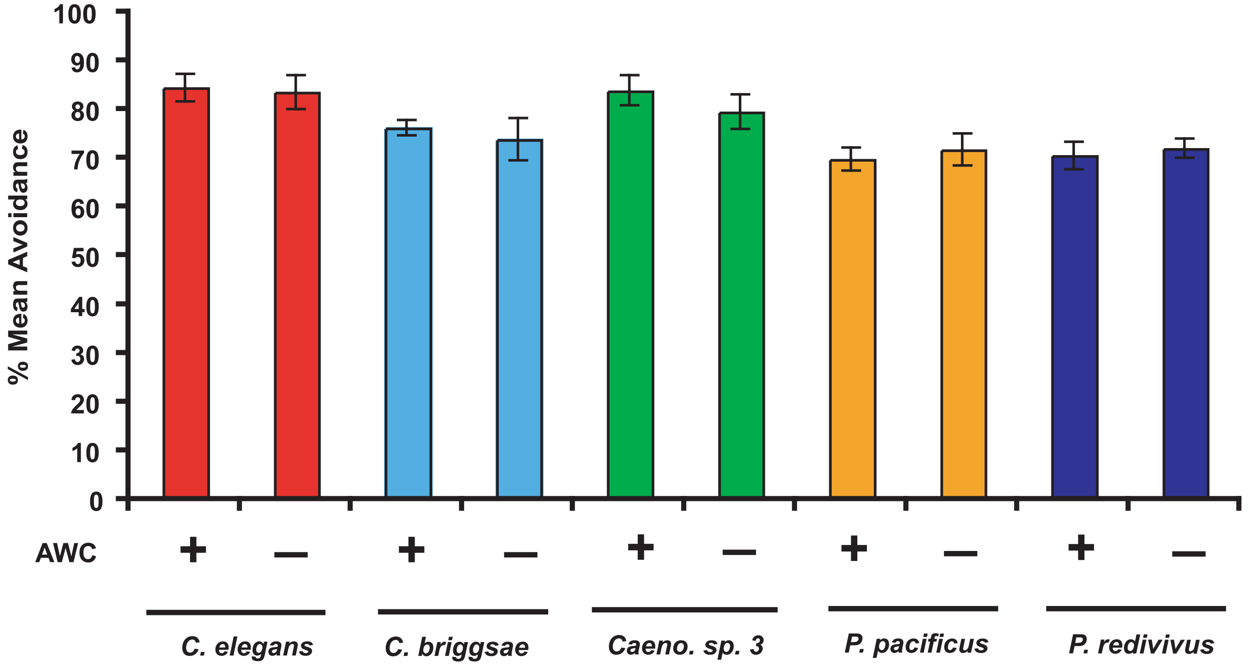

Supplementary Figure S3

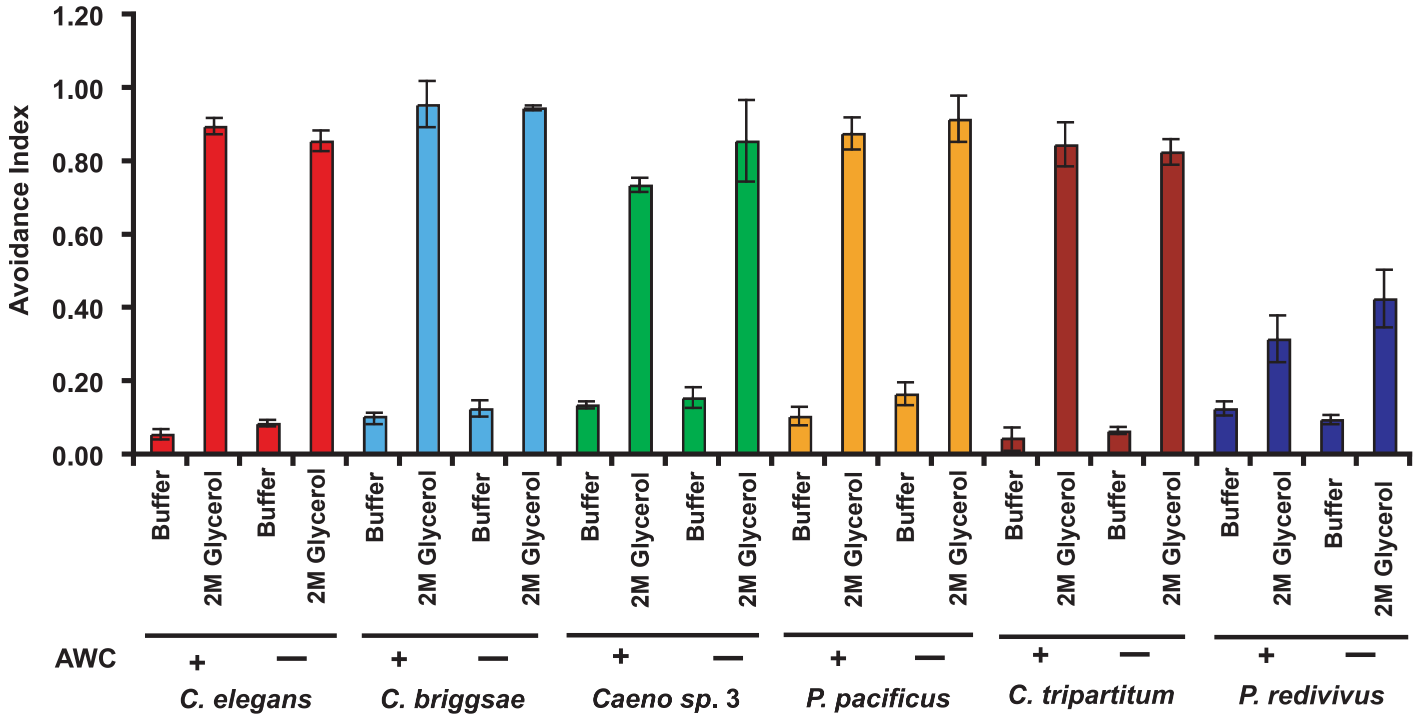

Supplementary Figure S4
